# Pancreatic Fat is not significantly correlated with β-cell Dysfunction in Patients with new-onset Type 2 Diabetes Mellitus using quantitative Computed Tomography

**DOI:** 10.7150/ijms.46395

**Published:** 2020-07-02

**Authors:** YX Li, YQ Sang, Yan Sun, XK Liu, HF Geng, Min Zha, Ben Wang, Fei Teng, HJ Sun, Yu Wang, QQ Qiu, Xiu Zang, Yun Wang, TT Wu, Peter M. Jones, Jun Liang, Wei Xu

**Affiliations:** 1 Graduate School of Bengbu Medical College, Bengbu, Anhui, China.; 2 Department of Endocrinology, Xuzhou Central Hospital, Xuzhou Institute of Medical Sciences, Xuzhou Clinical School of Nanjing Medical University, Affiliated Hospital of Medical School of Southeast University, Jiangsu, China.; 3 Department of Endocrinology, Affiliated Hospital of Nanjing University of Chinese Medicine, Jiangsu Province Hospital of Chinese Medicine, Jiangsu, China.; 4 Diabetes Research Group, Division of Diabetes & Nutritional Sciences, School of Medicine, King's College London, London, UK.

**Keywords:** Pancreatic fat, Quantitative computed tomography, Onset type 2 diabetes mellitus, β-cell function, Insulin resistance

## Abstract

**Objective:** Type 2 diabetes mellitus (T2DM) is a chronic condition resulting from insulin resistance and insufficient β-cell secretion, leading to improper glycaemic regulation. Previous studies have found that excessive fat deposits in organs such as the liver and muscle can cause insulin resistance through lipotoxicity that affects β-cell function. The relationships between fat deposits in pancreatic tissue, the function of β-cells, the method of visceral fat evaluation and T2DM have been sought by researchers. This study aims to elucidate the role of pancreatic fat deposits in the development of T2DM using quantitative computed tomography (QCT), especially their effects on islet β-cell function.

**Methods:** We examined 106 subjects at the onset of T2DM who had undergone abdominal QCT. Estimated pancreatic fat and liver fat were quantified using QCT and calculated. We analysed the correlations with Homeostatic Model Assessment of Insulin Resistance (HOMA-IR) scores and other oral glucose tolerance test-derived parameters that reflect islet function. Furthermore, correlations of estimated pancreatic fat and liver fat with the area under the curve for insulin (AUC_INS_) and HOMA-IR were assessed with partial correlation analysis and demonstrated by scatter plots.

**Results:** Associations were found between estimated liver fat and HOMA-IR, AUC_INS_, the modified β-cell function index (MBCI) and Homeostatic Model Assessment β (HOMA-β). However, no significant differences existed between estimated pancreas fat and those parameters. Similarly, after adjustment for sex, age and body mass index, only estimated liver fat was correlated with HOMA-IR and AUC_INS_.

**Conclusions:** This study suggests no significant correlation between pancreatic fat deposition and β-cell dysfunction in the early stages of T2DM using QCT as a screening tool. The deposits of fat in the pancreas and the resulting lipotoxicity may play an important role in the late stage of islet cell function dysfunction as the course of T2DM progresses.

## Introduction

T2DM is a metabolic disease characterized by chronic hyperglycaemia that is related to both insulin resistance and defective insulin secretion 1. Previous studies have shown that oxidative stress, low-grade chronic inflammation and activation of the renin-angiotensin system (RAS) all accelerate the development of T2DM 2-4. With the emergence of unhealthy lifestyles characterized by sedentary behaviours and overnutrition, the increase in metabolic disorders caused by fatty acid spill-over has become a focus of research.

Many studies have demonstrated the contribution of lipotoxicity to the development of T2DM, as the incidence of obesity and metabolic syndrome has increased. Previous study showed that in the non-diabetic state and in subjects with impaired glucose tolerance, circulating endogenous free fatty acids (FFAs) are not independently associated measures of β-cell function, and do not predict deterioration of glucose tolerance 5. Interestingly, many studies have found that FFAs are involved in the development of insulin resistance. When the level of FFAs in the blood increases, the excessive supply of FFAs and lipids exceeds adipose tissue adaptation, causing adipocyte insulin resistance and lipid accumulation in non-adipose tissues, such as the liver, muscle and pancreatic β cells as well as around the heart, and causing systemic or local dysfunction 6. It has been reported that ectopic fat deposition, such as lipid deposition in the liver, can increase peripheral insulin resistance 7- 10. There are clear methods, such as liver enzyme tests, magnetic resonance imaging (MRI), and quantitative computed tomography (QCT), that can be used to evaluate the degree of liver fat deposition. However, the effects of pancreatic tissue fat deposition on metabolism, insulin resistance and islet cell function have yet to be clearly investigated.

Schaefer first described pancreatic fat infiltration and reported the relationship between human body weight and pancreatic fat content in 1926 11. In 1933, Ogilvie autopsied 19 deceased patients and found differences in their pancreatic exocrine glands. The degree of fat increase, the predominant prevalence of obesity and the difference in pancreatic fat content between obese and non-obese individuals led to the development of the concept of "pancreatic lipomatosis" 12. Catanzaro R et al. proposed the concept of non-alcoholic fatty pancreas disease (NAFPD) in 2016 13. The role of pancreatic steatosis in the pathogenesis of T2DM is currently under discussion, as different views have emerged. It is unclear whether lipid accumulation in the pancreas contributes to β-cell dysfunction because to date, no effective enzymology or imaging method has been found that can easily and accurately assess pancreatic fat deposition.

Imaging studies are expected to produce reliable information regarding the amount and content of visceral fat. Indeed, QCT and MRI can accurately calculate the area and volume of intra-abdominal fat and are currently recognized as the gold standard for assessing adipose tissue distribution in the subcutaneous and abdominal areas 14. Hepatic and pancreatic fat content can be detected by proton magnetic resonance spectroscopy 15, computed tomography (CT) 16, and MRI 17, 18. However, MRI is not routinely used because it is time consuming and very expensive. QCT is corrected with a template, and the accuracy of the measurement is high. The measurement of fat is intuitive and accurate, and the accuracy of measuring abdominal fat with QCT is similar to that of MRI. There have been more studies on liver fat deposition 19-21 and fewer on the measurement of pancreatic fat deposition by QCT 22. We are interested in whether QCT can be used as an economical, efficient and accurate research tool to assess the extent of pancreatic fat deposition and its relationship to islet β-cell function.

The present study was designed to analyse the correlation between fat deposition in two organs and islet cell function in patients with newly diagnosed T2DM by quantifying the fat content of the liver and pancreas with QCT to determine the associations and clinical significance.

## Methods

### Study population

This study was carried out during the period from Feb 2016 to Jan 2019 at the Endocrinology Department of Xuzhou Central Hospital. A total of 106 subjects with newly diagnosed T2DM included 45 females and 61 males were selected. Ethics approval was obtained from the Ethical Committees of Xuzhou Central Hospital, and the study was conducted in accordance with the principles of the Declaration of Helsinki. Written informed consent was obtained from all participants.

The diagnostic criteria for diabetes were based on the 2014 American Diabetes Association (ADA) criteria. Patients with new-onset T2DM had a plasma fasting glucose level > 7.0 mmol/L or 2 h oral glucose tolerance test (OGTT) values >11.1 mmol/L without a previous diabetes diagnosis.

Subjects were excluded from this study if they had any of the following conditions: (1) hepatic or pancreatic disease, including inflammation, tumour, or autoimmune disease; (2) other malignant tumours; (3) acute complications of diabetes, including diabetic hyperosmolar coma, ketoacidosis, lactic acidosis, or hypoglycaemic coma; (4) active or chronic infection or inflammatory disease; (5) severe liver and kidney dysfunction; (6) use of medicine that affects blood or urine glucose levels; (7) acute cardio-cerebral vascular accident; or (8) pregnancy.

### Anthropometric measurements

Height and weight were measured by trained nurses with standardized and calibrated scales and stadiometers. Body mass index (BMI) was calculated with Microsoft Excel as weight in kilograms divided by the square of the height in metres. Waist circumference (WC) was measured midway between the superior border of the iliac crest and the lowest rib. Neck circumference (NC) was measured in the thinnest part of the neck below the throat. Systolic and diastolic blood pressures (SBP and DBP) were measured by trained nurses using a standard mercury sphygmomanometer after the subject had rested for at least 5 min. After an overnight 12-h fast, all subjects underwent routine biochemistry testing, including glycosylated haemoglobin (HbA1c), total cholesterol (TC), triglyceride (TG), high-density lipoprotein (HDL) and low-density lipoprotein (LDL).

### Beta cell function and insulin resistance

To estimate β-cell function (BCF) and insulin resistance (IR), several indices were used. A total of 106 subjects underwent a 2-h, 75-g OGTT during a screening visit. Serum insulin, C-peptide and plasma glucose levels were measured at every time point with blood samples drawn at 0, 30, 60 and 120 min after the subjects consumed the glucose. The areas under the curves (AUC), which were calculated using the trapezoid rule, for glucose and insulin were used to represent the total secretory response over the entire OGTT period. The modified β-cell function index (MBCI) was calculated as follows: (Fasting insulin (Fins)×fasting plasma glucose (FPG))/(120 min plasma glucose (Glucose_120min_)+60 min plasma glucose (Glucose_60min_)-2FPG) 23. IR was assessed by the Homeostatic Model Assessment of Insulin Resistance (HOMA-IR) (HOMA-IR=(Fins×FPG)/22.5). The insulin secretion index: Homeostatic Model Assessment β (HOMA-β) was calculated as 20×fasting C-peptide (FCP)/(FPG-3.5). ΔI/ΔG was calculated as the insulin increase at 30 min above the basal level divided by the corresponding glucose increase (Ins_30min_-Fins)/(Glucose_30min_ - FPG).

### Quantitative computed tomography

**Figure 1:**
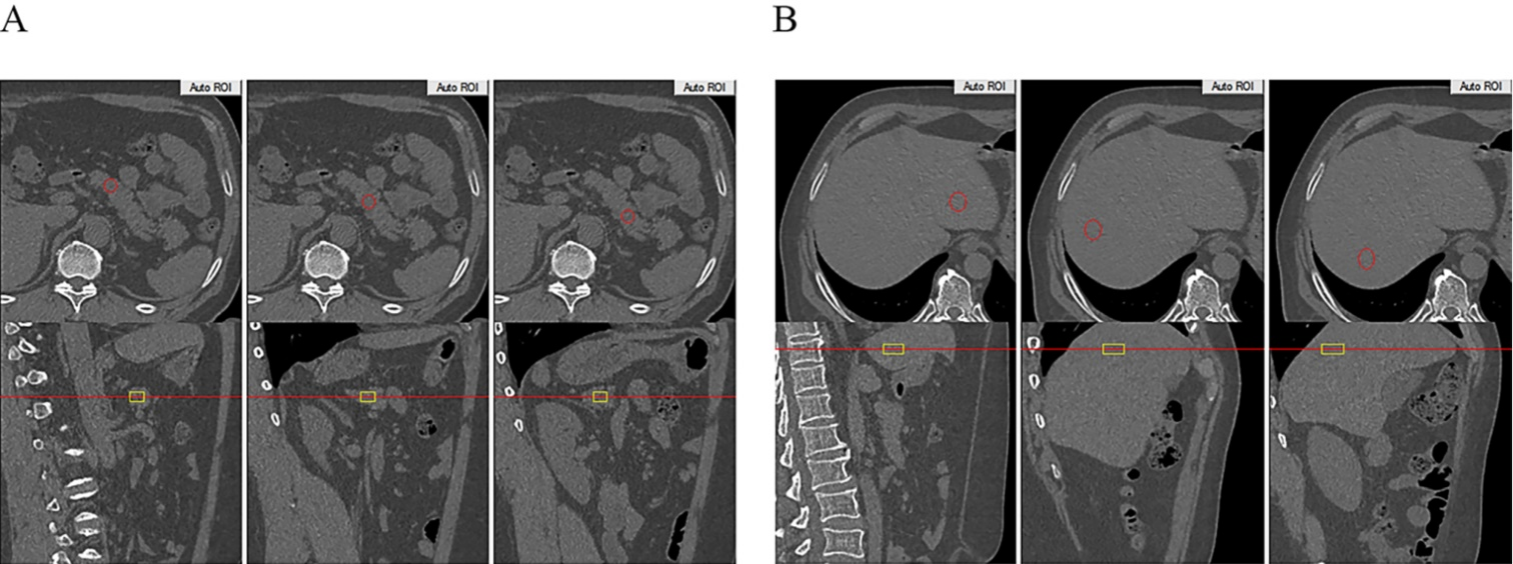
** QCT measurement of pancreatic and liver fat content. A:** Head, body and tail of the pancreas to measure pancreatic fat content. **B:** Left lobe, anterior segment of the right lobe and the posterior segment of the right lobe of the liver to measure liver fat content.

The subjects underwent routine CT scanning of the upper abdomen using a scanner (GE OPTIMA 680) with a QCT phantom (Mindways, Austin, TX, USA). The scanning parameters were as follows: tube current, 250 mA; tube voltage, 120 kvp; and section thickness, 1 mm. The CT scanning information was transmitted to the QCT Pro 3D spine module software workstation. For quantitative measurement of pancreatic fat, two experienced radiologists independently placed three round regions of interest (ROIs) manually in the pancreatic head, body, and tail in different QCT sections. Care was taken to not include intra-pancreatic vessels and pancreatic ducts. Each of the three ROIs had an area of approximately 100-150 mm^2^ (Figure 1A). For quantitative measurement of hepatic fat, ROIs were placed on the section at which the right branch of the portal vein enters the liver. Three circular ROIs covering an area of approximately 290 mm^2^ were drawn into the left lobe, the right anterior lobe and the right posterior lobe. Obvious blood vessels and bile ducts were avoided (Figure 1B). Using the export function of the QCT Pro software database, the field uniformity correction (FUC), the bone mineral density (BMD) value of three measurements and the corresponding calibration slope were derived.

QCT measurements of hepatic or pancreatic tissue using the Mindways spine module software were converted into percentage fat measurements using a published method for measuring liver fat and pancreatic fat by Cheng X et al. and calculated using the following equation 20-22:


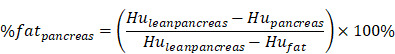



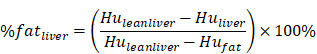


where HU_pancreas_ and HU_liver_ are the radiodensity measurements in the pancreatic and hepatic ROIs, and the values were found by converting the BMD measurements from the Mindways scan analysis software to radiodensity values using the QCT Pro scan calibration data. HU_fat_ and HU_leanliver_ and HU_leanpancreas_ are the radiodensity values for 100% fat and fat-free hepatic tissue and fat-free pancreatic tissue, respectively, and their values were found by representing fat and fat-free tissue in terms of their basis set equivalent densities of water (H2O) and dipotassium hydrogen phosphate (K2HPO4) and adjusting for tube voltage and person-to-person differences in beam hardening using scan calibration data from the QCT Pro software.

### Statistical analysis

All subjects were categorized into trisections of estimated pancreas fat and liver fat (T1, T2, T3). The data are presented as medians (interquartile range) or means ± standard deviations depending on the data distribution. We used ANOVA to explore the differences between groups. Correlations of estimated pancreas fat and liver fat with clinical parameters reflecting islet function or insulin resistance were assessed with Pearson's correlation, and those correlations were adjusted for sex, age, BMI (and HOMA-IR or WC) using partial correlation. Linear regression analysis was performed to show the correlation between islet function and estimated liver fat. The trend of AUC_INS_ and HOMA-IR changing with estimated pancreas fat or/and liver fat was shown using scatter plots. *P* values less than 0.05 were considered significant. SPSS statistical software version 22.0 was used for data management and statistical analyses.

## Results

### Comparison of new-onset T2DM characteristics according to estimated pancreas fat

A total of 106 subjects included 45 females and 61 males participated in the study. The baseline characteristics of the study subjects according to estimated pancreas fat tertiles are presented in Table 1. As the estimated pancreas fat tertile increased, patients were more likely to be older. The serum TC content increased across the estimated pancreas fat tertiles (*P* for trend<0.05). There was no significant trend or no trend of increase or decrease between estimated pancreas fat tertiles and WC, NC, BMI, SBP, DBP, serum insulin, C-peptide, plasma glucose, TG, HDL, LDL, HbA1c.

### Comparison of new-onset T2DM characteristics according to estimated liver fat

The baseline characteristics of the study subjects according to estimated liver fat tertiles are presented in Table 2. As the estimated liver fat tertile increased, patients were more likely to be younger. BMI (*P* for trend =0.03), Fins (*P* for trend=0.003), 120-min insulin (Ins_120min_) (*P* for trend=0.006), and FCP (*P* for trend=0.004) levels increased across estimated liver fat tertiles. There was a trend of increase between estimated liver fat tertiles and WC or NC, but this trend was not statistically significant. We could not find increasing or decreasing linear relationships between estimated liver fat tertiles and blood lipids.

### Estimated pancreas fat or liver fat and BCF

**Figure 2:**
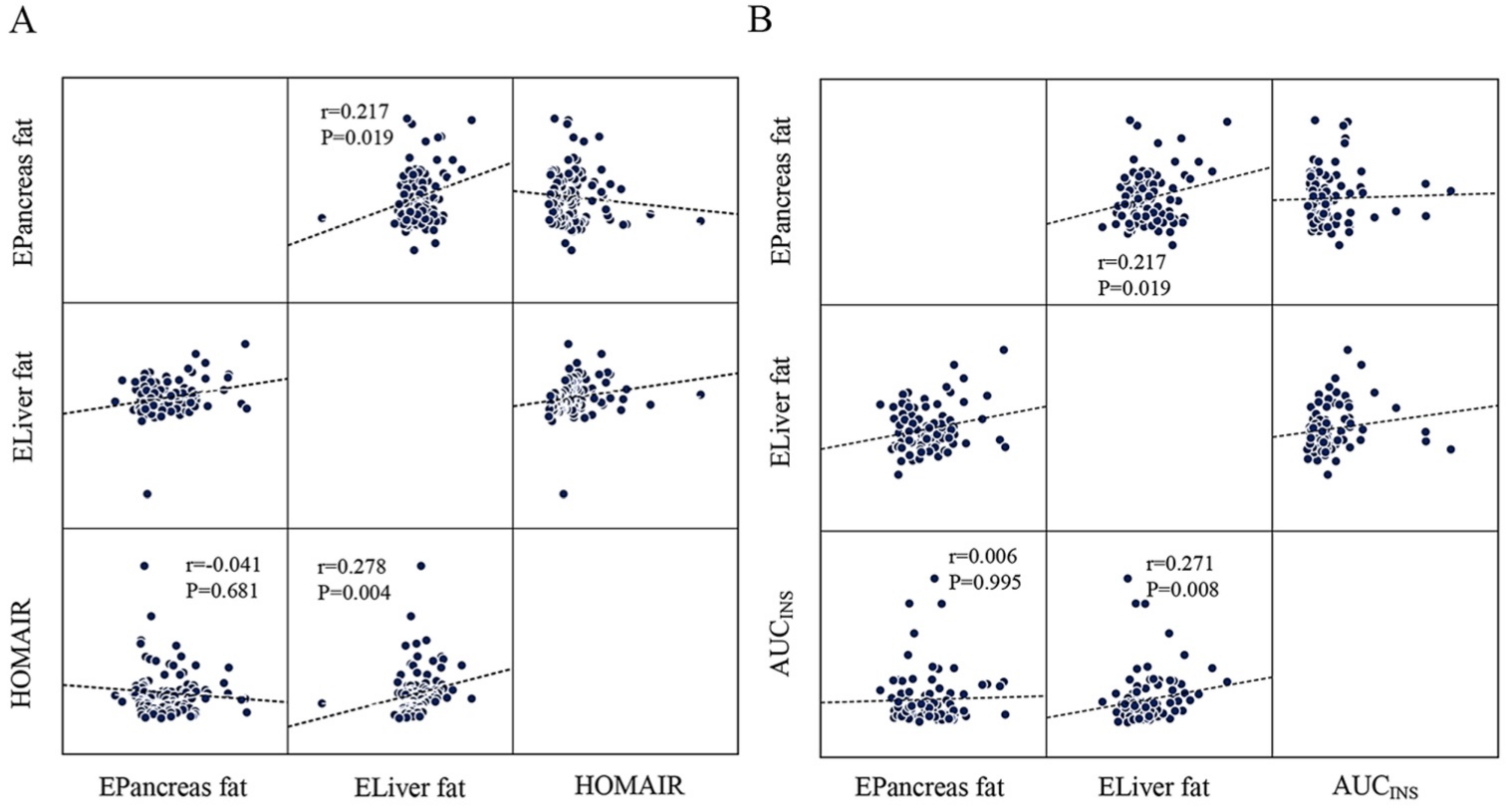
The scatter plots showing the correlation between HOMA-IR, AUC_INS_ and estimated pancreas fat or liver fat with the result of partial correlation analysis (controlling for sex, age and BMI). **A:** Estimated liver fat was positively correlated with HOMA-IR (*r*=0.278, *P*=0.004). No significant correlation was found between estimated pancreatic fat and HOMA-IR (*P*=0.681). **B:** Negative correlation of estimated liver fat with AUC_INS_ (*r*=0.271, *P*=0.008) and is significant compared to estimated pancreatic fat. (*P*=0.995). A positive correlation was found between estimated pancreatic fat and estimated liver fat (*r*=0.217, *P*=0.019).

AUC_INS_ (*r*=0.237, *P*=0.021), MBCI (*r*=0.297, *P*=0.004), and HOMA-β (*r*=0.267, *P*=0.006) were positively correlated with estimated liver fat in the Pearson correlation analysis. However, all correlations lost significance for estimated pancreas fat (Table 3). Linear regression analysis indicated that HOMA-β (*P*=0.014), AUC_INS_ (*P*=0.032), and MBCI (*P*=0.024) remained significantly associated with estimated liver fat (Table 4). Furthermore, AUC_INS_ was associated with estimated liver fat in the partial correlation analysis (*r*=0.271, *P*=0.008). We could not find a significant correlation between estimated pancreas fat and AUC_INS_ after adjustment for sex, age and BMI (Figure 2A). No significant correlations existed between AUC for glucose or ΔI/ΔG and estimated pancreas fat or liver fat in the Pearson correlation analysis (Table 3). The results regarding estimated liver fat and AUC_INS_ and HOMA-β remained significant after accounting for sex, age, BMI and HOMA-IR (or WC) (Supplementary Table 1, Supplementary Table 2).

### Estimated pancreas fat or liver fat and IR

IR as calculated by the HOMA-IR was positively correlated with estimated liver fat in the Pearson correlation analysis (*r*=0.311, *P*=0.001) (Table 3). Moreover, partial correlation analysis between estimated liver fat and HOMA-IR showed a correlation coefficient of *r*=0.278 (*P*=0.004) (Figure 2B). No significant correlations were found between estimated pancreas fat and HOMA-IR in either the Pearson or partial correlation analysis (Table 3, Figure 2B).

### Others

In the whole cohort, a positive correlation was found between estimated pancreas fat and estimated liver fat in the partial correlation analysis (*r*=0.217, *P*=0.019) (Figure 2B).

## Discussion

The present study analysed the association between fat deposition in the pancreas and islet cell function in patients with newly diagnosed T2DM by quantifying the fat in the pancreas using QCT. First, significant differences could be found in the estimated liver fat as well as in parameters of the BCF and IR. However, our study also provided an interesting perspective, showing that pancreatic fat in particular did not influence islet cell function in newly diagnosed T2DM patients. To the best of our knowledge, this is the first report to show the correlation of islet cell function and pancreatic fat deposition using QCT.

The excessive accumulation of fat in non-adipose tissues is a reported risk factor for IR in liver and skeletal muscle, contributing to the development and progression of diabetes 24-27. A theory has been proposed in which excess FFAs from the highly insulin-resistant visceral adipose tissue (VAT) spill into the liver, leading to excess hepatic adiposity and increased insulin resistance 28. Fat deposition in liver tissue leads to a decrease in the ability of insulin to suppress the production of glucose and the level of VLDL 29, 30. As the understanding of the mechanism of fat spill-over deepens, another theory proposed that increased intrahepatic lipid accumulation leads to increased export of VLDL-triglycerides from the liver, which subsequently deposit in the pancreas, contributing to β-cell failure 31. Fat deposition in the pancreas causes local chronic low-grade inflammation leading to islet cell inflammation and dysfunction 32- 34. Fat deposition in the pancreas could potentially have a destructive influence on insulin-producing β cells, both directly through lipotoxic mechanisms mediated by the release of FFAs and indirectly through the activation of inflammatory pathways 35, 36.

Given the interest in the role of excessive pancreatic fat deposition in the pathogenesis of T2DM, there have been few previous studies on the relationship between pancreatic fat and pancreatic endocrine function. One study found that pancreatic fat was a stronger determinant of impaired insulin secretion in subjects with prediabetes 37. Lu et al. found that pancreatic fat content is associated with β-cell function in Chinese T2DM subjects 38. Tushuizen et al. also concluded that the pancreatic fat content was inversely associated with β-cell function in nondiabetic subjects 39. In contrast, some studies have found no association of NAFPD with β-cell function. Researchers from Hong Kong used MRI as a research tool to confirm the lack of a significant correlation between NAFPD and β-cell function after adjusting for hepatic fat content and BMI 40. Similar findings were also obtained in another study in which research could not establish an association between pancreatic fat content and β-cell function in individuals with impaired glucose metabolism 41. Wicklow et al. also found that pancreatic triglyceride content, as measured by magnetic resonance spectroscopy (MRS), is not associated with β-cell function in youth with new-onset T2DM 42.

The results we obtained using QCT as a research tool were consistent with the latter study mentioned above. Our study showed that significant differences could be found in estimated liver fat as well as in the parameters of the BCF and IR. HOMA-IR was positively correlated with estimated liver fat. Interestingly, a positive correlation was found regarding estimated liver fat and AUC_INS_, MBCI, and HOMA-β. However, we could not establish a relationship between pancreatic fat content and β-cell function. These previous findings and our concurring data lead us to conclude that pancreatic fat is not associated with BCF in individuals with prediabetes and new-onset T2DM 41, 42. This provides us with more insight into the pathogenesis of T2DM, which is characterized by insulin resistance and β-cell dysfunction. There are two phenomena, glucotoxicity and lipotoxicity, which have been postulated to harm β cells 43. Subsequently, the term "glucolipotoxicity" was coined in recognition of the interrelationship between glucotoxicity and lipotoxicity 43, 44. Many findings support the hypothesis that glucotoxicity is a necessary initial condition of lipotoxicity 45, 46. Long-term lipotoxicity has a more important effect on the inhibition of insulin release 47. The deleterious effect of pancreatic fat on β cells may be permissive to hyperglycaemia in the early stages of T2DM. The deposits of fat in the pancreas and the resulting lipotoxicity may play an important role in the late stage of islet cell dysfunction as the course of T2DM progresses. The influence of glucotoxicity and lipotoxicity on β-cell function and their effects on the pathogenesis of T2DM remain to be further explored.

The association of pancreatic fat and β-cell function was not found in new-onset T2DM. This may be explained by pathophysiological factors, which are more likely to account for the findings. It has been theorized that once T2DM occurs, the effect of other factors superimposing pancreatic steatosis may account for the gradual decline in β-cell function. That is, due to many deleterious cascades, including inflammation, oxidative stress, apoptosis and hypoperfusion of islets, the rate of deterioration of β-cell function is not disproportionate to pancreatic fat accumulation 39. Additionally, we cannot discriminate between intra- and inter-islet fat accumulations. There may be different mechanisms for the effects of pancreatic exocrine tissue fat deposition and pancreatic islet cell fat deposition on pancreatic islet function. Our observation was found only at the onset of T2DM in individuals and not in other stages of T2DM. Our results support this hypothesis: fat deposits in the liver, an important organ involved in lipid metabolism, play a primary role in IR in early diabetes. The role of the pancreas may be due to the specificity of the tissue structure (there is no specific cell that can store a large amount of fat), and the effect of pancreatic fat deposition on islet cell function cannot be equal to the effect of liver fat deposition on islet cell function.

Abnormal accumulation of fat in hepatocytes cause fatty liver has been clear, but the deposition of fat in the pancreas is currently unclear. Recent studies have found only that fat deposition in pancreatic tissue occurs mostly between lobules 48,49 or that triglycerides are found inside islet cell sediment 50. The development and functional effects of pancreatic fat deposition are still in the exploration stage. Our team has also made some meagre efforts in the research direction of the mechanism of pancreatic fat deposition. We paid more attention to the pathophysiological mechanism of fat deposition inside the islet because its effect on islet function may exceed the role of fat deposition in pancreatic exocrine tissue.

Our previous studies have confirmed the presence of stellate cells within the islets (ISCs), which are identical in the expression of retinoid-containing fat droplets 51, 52, 53, 54,55. A loss of oil red O-stained fat droplets was associated with the activation of diabetic ISCs, and we speculate whether the transfer of lipid droplets from the inside to the outside of the ISCs leads to local fat deposition and the inhibition of normal β-cell function. We are looking for a process of fat droplet metabolism in ISCs, hoping to find a specific target cell for pancreatic fat deposition studies. The stellate cells present in pancreatic exocrine tissues exhibit biological behaviour similar to that in pancreatic islet stellate cells in fat droplet metabolism. We speculate that pancreatic stellate cells (PSCs) may be the target cell for fat deposition in pancreatic exocrine tissues.

Other studies have found striking ethnic differences in the relationship between pancreatic fat and β-cell dysfunction. An inverse relationship between pancreatic fat and insulin secretion was seen in Hispanic adults with mild obesity rather than in black or white adults 56 and African American minority adolescents with prediabetes rather than Latinos 57. This discrepancy may be due to the differences in the populations studied and suggests a major influence of ancestral genes. However, our study targeted only a small population with new-onset T2DM in China and concluded that there was no correlation between pancreatic fat and β-cell function. The harmful effect of pancreatic fat on insulin secretion may be strengthened in persons who are genetically predisposed to develop T2DM. The racial or ethnic genetic difference in ectopic fat is still unknown and requires further exploration.

In addition to pathophysiological and population selection factors, it is possible that methodological factors also influenced our results. Imaging techniques such as ultrasound, MRI and CT can be used to measure regional adipose tissue *in vivo* 18, 58, 59. Although MRI is free from radiation, its application is still limited by its high cost and long scanning time. Compared to MRI, CT is convenient and well tolerated by the public. Adipose tissue can be measured quickly and accurately using QCT 60. Previous studies have reported the application of QCT for the determination of ectopic fat deposition in the liver 20, 61. An animal study showed that both QCT and MRI measurements of goose liver fat were accurate and reliable compared with chemical methods *in vitro* 19. However, only one study has been found on the measurement of pancreatic fat by QCT 22, but no studies have explored the relationship between pancreatic fat quantification with QCT and islet β-cell function. Our present study quantified pancreatic and hepatic fat by QCT. No association between estimated pancreatic fat and islet cell function was found in patients with new-onset T2DM. In addition, we may attribute this to imaging technique. Yao WJ et al. reported that systematic differences between QCT and chemical-shift-encoded magnetic resonance imaging exist, and the result strongly suggests a good correlation between the two measurements 22. Our results clearly supported that there is no significant correlation between pancreatic fat deposition and β-cell function in the early stages of T2DM using QCT. The method for assessing pancreatic fat deposition using QCT will be further improved in the subsequent assessments of clinical tests.

Our study had several limitations. In this study, blood glucose was measured at only 4 time points, so the model of glucose-stimulated insulin secretion proposed by Mari et al. 62 could not be established. The use of HOMA-β to respond to insulin secretion may affect the results of the experiment. Although various functions to assess β-cell function and insulin resistance were used, a gold-standard technique such as a clamp study or minimal modelling of intravenous glucose challenge was not used. Furthermore, the evaluation of the fat content was not done with gold-standard methods, and the studied subjects were exposed to the radiation burden. Finally, the sample size was relatively small and further expansion of the sample size, long-term follow-up observation of patients and specialized animal *in vivo* experiments to determine molecular mechanisms still need further exploration to elucidate further insights into the pathogenesis of pancreatic fat deposition.

Given the above findings, we conclude that ectopic lipid deposition in the liver is related to IR and β-cell dysfunction. There was no significant correlation between pancreatic fat deposition and β-cell function in 106 adults with newly diagnosed T2DM. More meaningfully, this is first report to use QCT as a method to evaluate the correlation of islet cell function and pancreatic fat deposition. Long-term research is needed to gain more insight into the contributing role of ectopic fat in the pancreas to the progressive decline in β-cell function.

## Supplementary Material

Supplementary tables.

## Acknowledgement and declarations

This work was supported by generous grants from the Natural Science Foundation of Jiangsu Province (BK20171171), the Xuzhou Science and Technology Bureau project (KC16SH110), the National Nature Science Foundation of China (NSFC- 81803908). The author gratefully acknowledges the support of K.C.Wong Education Foundation, Hong Kong.

### Ethics approval and consent to participate

This study was reviewed and approved by the Ethical Committees of Xuzhou Central Hospital. Written informed consent was obtained from all of the participants.

### Availability of data and material

Please contact the corresponding author with data requests.

### Authors' Contributions

All authors have approved the final version of the manuscript. Wei Xu is responsible for the integrity of the work as a whole. YX Li, YQ Sang, Yan Sun, XK Liu, HF Geng and Min Zha contributed equally to this work.

## Figures and Tables

**Table 1:** Clinical and biochemical characteristics of new-onset T2DM according to estimated pancreatic fat tertiles

	Estimated pancreas fat by QCT			
Variables	T1	T2	T3	P for trend
Age (years)	41.69±14.93	47.85±10.49	48.05±12.86	0.031
WC (cm)	102.48±17.21	93.06±10.98	95.30±7.93	0.123
NC (cm)	41.43±5.29	36.32±3.63	38.70±3.06	0.063
BMI (kg/m^2^)	28.52±6.81	26.55±5.76	27.16±5.23	0.325
SBP (mmHg)	134.03±9.81	131.79±13.18	128.16±13.77	0.774
DBP (mmHg)	79.85±8.69	82.92±8.61	79.30±8.11	0.135
*Fins (µIU/mL)	8.3 (5.46~13.47)	7.91 (5.40~12.24)	7.11 (3.80~14.19)	0.964
*Ins_30min_ (µIU/mL)	16.65 (11.50~34.51)	21.26 (8.79~27.85)	13.06 (7.34~34.60)	0.484
*Ins_60min_ (µIU/mL)	26.91 (14.50~49.71)	30.27 (17.22~49.05)	17.83 (10.49~58.08)	0.289
*Ins_120min_ (µIU/mL)	25.14 (18.10~48.63)	31.72 (15.30~41.02)	23.26 (10.59~58.63)	0.973
FCP (ng/mL)	3.02±1.83	2.51±1.25	2.83±1.26	0.166
CP_30min_ (ng/mL)	4.12±2.21	4.09±3.69	3.63±2.35	0.728
CP_60min_ (ng/mL)	5.32±2.93	5.70±4.04	4.73±3.08	0.899
CP_120min_ (ng/mL)	6.23±3.79	7.15±6.38	6.59±3.68	0.439
FPG (mmol/L)	10.82±3.87	9.70±3.87	10.02±3.27	0.329
Glucose_30min_ (mmol/L)	13.97±3.73	13.75±4.38	14.80±4.28	0.464
Glucose_60min_ (mmol/L)	17.63±3.70	17.20±5.18	18.31±4.66	0.413
Glucose_120min_ (mmol/L)	17.82±5.85	16.46±6.28	18.98±5.36	0.097
HbA1c (%)	10.65±3.10	9.14±2.67	10.12±2.67	0.842
TC (mmol/L)	4.82±1.32	5.03±0.98	5.16±0.97	0.043
TG (mmol/L)	2.70±2.11	2.43±1.38	2.87±2.42	0.737
HDL (mmol/L)	1.05±0.24	1.09±0.21	1.01±0.18	0.139
LDL (mmol/L)	3.04±0.92	3.22±0.70	3.32±0.88	0.823

Data are shown as medians (interquartile range) or means ± standard deviations depending on the data distribution. WC, waist circumference; NC, neck circumference; BMI, body mass index; SBP, systolic blood pressure; DBP, diastolic blood pressure; Fins, fasting insulin; Ins_30min_, 30 min insulin; Ins_60min_, 60 min insulin; Ins_120min_, 120 min insulin; FCP, fasting C-peptide; CP_30min_, 30 min C-peptide; CP_60min_, 60 min C-peptide; CP_120min_, 120 min C-peptide; FPG, fasting plasma glucose; Glucose_30min_, 30 min plasma glucose; Glucose_60min_, 60 min plasma glucose; Glucose_120min_, 120 min plasma glucose; HbA1c, glycosylated haemoglobin; TC, total cholesterol; TG, triglyceride; HDL, high-density lipoprotein; LDL, low-density lipoprotein.

**Table 2:** Clinical and biochemical characteristics of new-onset T2DM according to estimated liver fat tertiles

	Estimated liver fat by QCT			
Variables	T1	T2	T3	P for trend
Age (years)	50.33±11.82	47.28±12.51	39.68±13.13	<0.001
WC (cm)	93.76±7.42	97.52±15.47	100.78±15.18	0.107
NC (cm)	37.90±3.34	38.86±5.42	40.43±4.66	0.09
BMI (kg/m^2^)	26.17±5.40	26.99±7.07	29.19±4.99	0.03
SBP (mmHg)	130.74±11.68	133.32±14.96	130.11±10.63	0.826
DBP (mmHg)	79.64±8.40	82.00±7.98	80.27±9.31	0.750
*Fins (µIU/mL)	6.47 (3.78~10.71)	7.07 (4.09~12.40)	10.73 (7.05~14.73)	0.003
*Ins_30min_ (µIU/mL)	15.93 (8.08~23.86)	13.80 (8.36~28.86)	21.80 (11.69~37.05)	0.127
*Ins_60min_ (µIU/mL)	19.79 (11.66~38.46)	23.51 (10.91~36.26)	32.17 (15.94~63.22)	0.158
*Ins_120min_ (µIU/mL)	19.77 (13.09~33.30)	24.00 (14.92~43.32)	41.32 (18.76~68.35)	0.006
FCP (ng/mL)	2.31±1.35	2.70±1.59	3.32±1.31	0.004
CP_30min_ (ng/mL)	3.69±3.82	3.69±2.52	4.47±1.85	0.281
CP_60min_ (ng/mL)	4.65±4.09	5.25±3.49	5.79±2.57	0.194
CP_120min_ (ng/mL)	5.67±6.40	6.76±3.97	7.44±3.40	0.114
FPG (mmol/L)	10.57±3.56	10.17±4.05	9.91±3.42	0.445
Glucose_30min_ (mmol/L)	14.80±3.75	13.99±4.83	13.81±3.78	0.341
Glucose_60min_ (mmol/L)	18.24±4.52	17.25±5.18	17.73±4.01	0.661
Glucose_120min_ (mmol/L)	18.14±6.07	17.26±6.48	17.90±5.18	0.866
HbA1c (%)	10.67±2.99	9.72±3.01	9.73±2.55	0.177
TC (mmol/L)	5.03±1.11	4.98±1.19	5.02±1.02	0.978
TG (mmol/L)	2.63±2.05	2.17±1.20	3.24±2.50	0.191
HDL (mmol/L)	1.06±0.19	1.09±0.25	1.01±0.21	0.321
LDL (mmol/L)	3.15±0.81	3.28±0.92	3.16±0.81	0.955

Data are shown as medians (interquartile range) or means ± standard deviations depending on the data distribution. WC, waist circumference; NC, neck circumference; BMI, body mass index; SBP, systolic blood pressure; DBP, diastolic blood pressure; Fins, fasting insulin; Ins_30min_, 30 min insulin; Ins_60min_, 60 min insulin; Ins_120min_, 120 min insulin; FCP, fasting C-peptide; CP_30min_, 30 min C-peptide; CP_60min_, 60 min C-peptide; CP_120min_, 120 min C-peptide; FPG, fasting plasma glucose; Glucose_30min_, 30 min plasma glucose; Glucose_60min_, 60 min plasma glucose; Glucose_120min_, 120 min plasma glucose; HbA1c, glycosylated haemoglobin; TC, total cholesterol; TG, triglyceride; HDL, high-density lipoprotein; LDL, low-density lipoprotein.

**Table 3:** Pearson correlation analysis between estimated pancreas fat or liver fat and clinical parameters reflecting islet function or insulin resistance

	Estimated pancreas fat by QCT		Estimated liver fat by QCT	
Variables	r	P	r	P
HOMA-IR	-0.033	0.738	0.311	0.001
AUC_PG_	0.031	0.765	-0.014	0.893
AUC_INS_	-0.045	0.664	0.237	0.021
MBCI	-0.131	0.217	0.297	0.004
ΔI/ΔG	-0.137	0.194	0.173	0.101
HOMA-β	0.020	0.836	0.267	0.006

HOMA-IR, Homeostatic Model Assessment of Insulin Resistance; AUC_PG_, AUC for plasma glucose; AUC_INS_, AUC for insulin; MBCI, modified β-cell function index; I, insulin; G, glucose; HOMA-β, homeostatic model assessment β.

**Table 4:** Linear regression analysis between estimated liver fat and islet function

	Beta	95%CI	t	P
Y=HOMA-β	0.016	0.003~0.029	2.503	0.014
Y=AUC_INS_	0.012	0.001~0.023	2.18	0.032
Y=MBCI	0.012	0.002~0.022	2.301	0.024

HOMA-β, homeostatic model assessment β; AUC_INS_, AUC for insulin; MBCI, modified β-cell function index; CI, confidence interval.
