# Supplementary material for: Pancreatic Fat is not significantly correlated with β-cell Dysfunction in Patients with new-onset Type 2 Diabetes Mellitus using quantitative Computed Tomography
Source: Int J Med Sci. 2020 Jul 2;17(12):1673–82. doi: 10.7150/ijms.46395 (PMC7378671; doi:10.7150/ijms.46395)
Supplement: Supplementary file 1 — Supplementary tables. [file ijmsv17p1673s1.pdf]

Supplement table 1 The partial correlation analysis between estimated pancreatic fat or liver fat and clinical parameters reflects islet function (controlling for sex, age, BMI and HOMA-IR).

	Estimated pancreatic fat by QCT		Estimated liver fat by QCT	
Variables	r	P	r	P
AUC_{PG}	0.022	0.838	-0.045	0.681
AUC_{INS}	0.095	0.385	0.289	0.007
MBCI	0.02	0.858	0.2	0.066
▵I/△G	-0.022	0.842	-0.16	0.143
HOMA-β	0.141	0.199	0.237	0.029

AUC_{PG}, AUC for plasma glucose; AUC_{INS}, AUC for insulin; MBCI, modified β-cell function index; I, insulin; G, glucose; HOMA-β, homeostatic model assessment β.

Supplement table 2 The partial correlation analysis between estimated pancreatic fat or liver fat and clinical parameters reflects islet function (controlling for sex, age, BMI and WC).

	Estimated pancreatic fat by QCT		Estimated liver fat by QCT	
Variables	r	P	r	P
AUC_{PG}	-0.024	0.874	-0.088	0.552
AUC_{INS}	0.058	0.696	0.31	0.032
MBCI	-0.033	0.826	-0.087	0.559
ΔI/ΔG	-0.136	0.355	-0.03	0.84
HOMA-β	0.098	0.508	0.247	0.009

AUC_{PG}, AUC for plasma glucose; AUC_{INS}, AUC for insulin; MBCI, modified β-cell function index; I, insulin; G, glucose; HOMA-β, homeostatic model assessment β.
